# Supplementary material for: A pan-cancer analysis of homeobox family: expression characteristics and latent significance in prognosis and immune microenvironment
Source: Front Oncol. 2025 Feb 6;15:1521652. doi: 10.3389/fonc.2025.1521652 (PMC11840236; doi:10.3389/fonc.2025.1521652)
Supplement: Supplementary Figure 1 — Mutations of each HOX gene in 32 cancers (TCGA, PanCancer Atlas) by cBioportal. [file DataSheet1.zip › Suppl.files/raw code.zip!/code/4.survival/survivalplot.zip!/survivalplot/HOXD11_OS.KIRP.pdf]

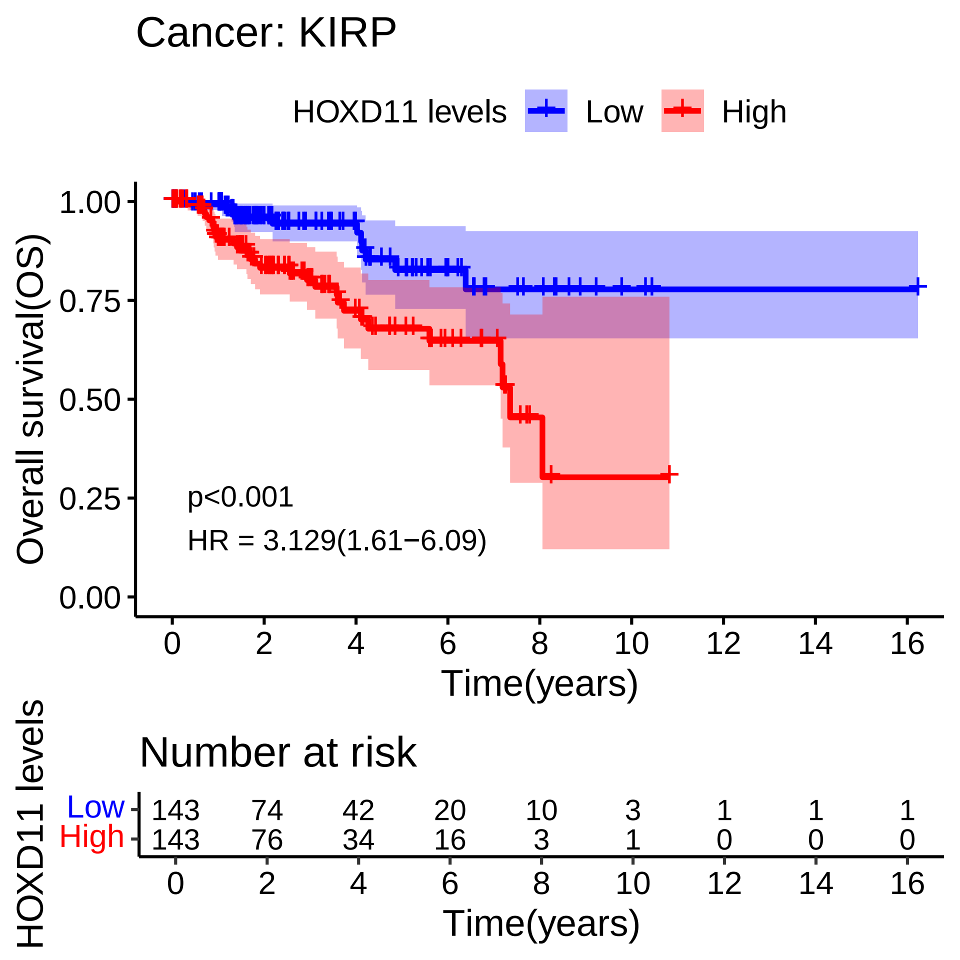

Cancer: KIRP
HOXD11 levels
Low
High
Overall survival(OS)
1.00
0.75
0.50
0.25
0.00
p<0.001
HR = 3.129(1.61−6.09)
0 2 4 6 8 10 12 14 16
Time(years)
Number at risk
HOXD11 levels
Low 143 74 42 20 10 3 1 1 1
High 143 76 34 16 3 1 0 0 0
0 2 4 6 8 10 12 14 16
Time(years)
